# At-Home Bioelectrical Impedance Analysis (BIA) Monitoring of Adult Females at Risk of Breast Cancer-Related Lymphedema: Nonrandomized One-Year Longitudinal Feasibility Study

**DOI:** 10.2196/73978

**Published:** 2025-07-24

**Authors:** Cheri Teranishi-Hashimoto, Monica Padilla, Yoomi Heo, Lori Bravi

**Affiliations:** 1 REHAB Hospital of the Pacific, Honolulu, HI, United States; 2 InBody BWA, 2550 Eisenhower Avenue, Suite C209, Trooper, PA, 19403, United States, 1 5627415507; 3 InBody Co, Ltd, Seoul, Republic of Korea; 4 Shirley Ryan Ability Lab, Chicago, IL, United States

**Keywords:** breast cancer-related lymphedema, home-based bioimpedance analyzer (BIA), extracellular water, remote monitoring, case report

## Abstract

**Background:**

Breast cancer, the most common type of diagnosed cancer in women worldwide, is often associated with the development of lymphedema as a treatment-related effect. Patients undergoing surgery, radiation, or chemotherapy present a higher risk for this side effect. Historically, patients are often not referred to rehabilitation for lymphedema management until the swelling is visible and has progressed, which reduces the chance of reversing the disease progression. Surveillance is key to identifying the earliest signs of breast cancer–related lymphedema, initiating treatment, reversing the disease process, and reducing the impact on function.

**Objective:**

The primary goal of this study was to assess the feasibility of monitoring fluid levels in patients with cancer using a bioimpedance analysis home device and to detect any relevant changes that may correlate with an increased risk of developing lymphedema. Remote monitoring by a clinician has not previously been possible, and a comparable bioelectrical water analyzer device has never been available to patients within the comfort of their homes.

**Methods:**

The study included 8 adult patients diagnosed with unilateral breast cancer who underwent unilateral lumpectomy or mastectomy, bilateral mastectomy, or reconstruction and were followed for 12 months. Patients also underwent radiation or chemotherapy as part of their treatment before the study and, in some cases, during participation in the study. Clinic visits were required every 3 months, with standard care treatment administered by the clinician, as well as daily fluid monitoring, using the extracellular to total body water (ECW/TBW) ratio obtained with the home device.

**Results:**

Preliminary findings from the 8 cases showed that daily monitoring with the home device is possible, and may aid in the detection of fluid changes due to interventions like radiation or chemotherapy; these changes typically subside after treatment, compared to a permanent fluid increase that may indicate lymphedema. While one participant developed lymphedema, there is insufficient data to generalize the feasibility of early detection using the home device. A significant difference between ECW/TBW ratio measurements taken in the morning and evening (*P* values<.016) was observed for 6 participants, with morning values being higher than evening ones. Additionally, 7 participants showed a higher ECW/TBW ratio in the affected arm compared to the unaffected arm. On average, the ratio between the two values was higher than 1, approximately 75% of the time. The daily monitoring empowered patients to take charge of their health, with more than half expressing a desire to continue using the home device beyond the end of the study period.

**Conclusions:**

This case report shows the feasibility of daily remote monitoring for patients at risk of developing BCRL using a home bioimpedance analysis device.

## Introduction

BCRL (Breast cancer-related lymphedema) is the most common complication arising after breast cancer surgery and treatment, characterized by abnormal swelling postsurgery or even years after treatment [1,2]. While lymphedema can be managed once detected, it remains a lifetime risk, for which patients should remain vigilant [3]. Early surveillance allows risk reduction through therapy interventions and reversal of the early-stage BCRL, particularly stages 0-1 as defined by the International Society of Lymphology. Data show that one in every five women with a history of breast cancer develop BCRL [4], most likely caused by surgical removal of lymph nodes or fluid circulation or radiotherapy-induced scarring of lymph nodes and surrounding circulation, leading to an imbalance in lymphatic fluid. Early diagnosis and precise evaluation are necessary to minimize edema progression and to prevent lymphatic drainage failure. Lymphedema is a chronic condition characterized by swelling from lymphatic fluid buildup and may be primary (hereditary or congenital) or secondary (caused by trauma such as surgery, radiation therapy, or infection). Recent studies have found that other factors, including predisposition to disease may increase the incidence of secondary lymphedema [5]. Early diagnosis and management through therapies (eg, manual drainage and compression bandages), compression garments, exercise, and limb elevation help control symptoms and prevent complications [6,7]. Standard clinical practice involves circumferential or volumetric measurements to monitor patients with cancer after surgery, which occur as often as monthly to every 3 or 6 months, depending on the treatment protocol at the clinic, and whether the patient is referred to therapy. However, subclinical (ie, not visible) lymphedema may go unnoticed as using tape measurement is not sensitive enough to detect such a slight increase in volume. Organizations such as the National Lymphedema Network, the International Society of Lymphology, and the National Accreditation Program for Breast Cancer Centers recommend that patients should be assessed before surgery and then monitored at regular intervals, although no gold standard measurement has been defined [2]. Furthermore, despite the growing awareness and prevalence of BCRL, a significant deficit remains in patient education and breast cancer treatment paradigm for early surveillance of lymphedema [8]. Often, patients are not proactively referred to therapy, but reactively once symptoms are already apparent, and have progressed from Stage 0 to Stage 1 [6,7,9]. Although invasive procedures such as new microsurgery and surgery interventions are being used, complete decongestive therapy remains the most common treatment for BCRL. In advanced stages of lymphedema, patients can develop fatty fibrosis, which may require liposuction procedures to remove the affected tissue [8].

Professional bioelectrical impedance analysis (BIA) devices have been previously used in clinical settings to aid in the detection of lymphedema [10] and to evaluate the effectiveness of provided treatment [11]. These devices send low-amplitude alternating currents through the body at different frequencies to measure the resistance (ie, impedance) to the flow of that current in the body water. These data allow estimation of the complete amount of water in the body (TBW, total body water), the water outside the cells (ECW, extracellular water), and the water inside the cells (ICW, intracellular water) can be estimated. The ECW/TBW ratio serves as an indication of inflammation or edema (ie, EI – Edema Index). The InBody BWA ON (InBody Co. Ltd., Seoul, Korea, via InBody BWA) device uses BIA to assess body composition, including hydration levels, fat, and muscle mass, segmentally, which may aid in lymphedema diagnosis. Like other BIA devices, it is easy to use, fast, reproducible, and in the case of the BWA ON, also portable. Regular measurements with home-use devices such as the BWA ON had not been available before; these tools may enable early lymphedema detection and management, reducing health care burden and quality-of-life costs associated with BCRL diagnosed in later stages. Real-time data measurements are shared with health care providers for customized consultations and prescriptions, evaluation of treatment effectiveness, as well as patient compliance, while also monitoring weight, muscle mass, and body fat percentage—further supporting prevention and management of lymphedema.

## Methods

### Participants

Eight adult female patients diagnosed with unilateral breast cancer who underwent surgery with lymph node removal 1‐12 months before study enrollment participated in the study. Exclusion criteria included bilateral cancer, as the aim was to detect fluid differences between the affected and unaffected arms; previous history of breast cancer; existing lymphedema diagnosis; and cellulitis or other active infection at the time of enrollment. Additionally, other exclusion criteria were pregnancy (as it could affect body composition and body fluids), being unable to provide informed consent, prisoners, and other vulnerable populations. As indicated by BIA testing, individuals with pacemakers were excluded. Inclusion criteria were: 18 years old or older, unilateral breast cancer diagnosis, and having undergone a procedure that would increase their risk of developing lymphedema. Patients undergoing bilateral mastectomy (prophylactic) and breast reconstruction could be included. Participants were required to have a phone so they could install the app and test themselves at home. There were no exclusions based on age, BMI, or comorbidities, unless these conditions (eg, kidney disease or heart failure) could affect fluid levels. Participants included in this initial report were tested at two recognized institutions: REHAB Hospital of the Pacific in Honolulu (Site 1) and Shirley Ryan AbilityLab in Chicago (Site 2).

### Ethical Considerations

The local institutional review board (IRB) at each site reviewed the study protocol, consent form, and all required details for human research. The IRB used at the REHAB Hospital of the Pacific was the University of Hawaii IRB (UH IRB, IRB2022-00055) and for the SRALAB in Chicago, the Northwestern University IRB was used (NU IRB, STRU00216604). No study-related activities were initiated before IRB approval was granted.

Informed consent approved by the IRB at each site was discussed with possible participants, and consent was obtained before any research activity was conducted. Possible participants were either referred to or contacted by the researcher or clinician if they met the inclusion criteria, or they reached out directly. Patients who might qualify for the study and required follow-up after unilateral breast cancer diagnosis and treatment were referred by their surgeons or oncologists. They could contact the site directly or be contacted by the researcher or therapist at the corresponding site, based on their preference. After receiving information about the study and what participation would entail, patients could schedule a clinic visit to complete enrollment. Patients referred to the sites for standard treatment or who had already been seen and had expressed willingness to participate in research, were approached by the therapist to determine if they qualified for the study, and details of the study were shared with them.

All questions regarding the informed consent and study activities were answered before consent forms were signed. As with any other research study, participants were allowed to withdraw from the study without any repercussions or without affecting treatment, if they were receiving any type of therapy related to their condition or risks.

Privacy and confidentiality were guaranteed in accordance with IRB-approved protocols and were clearly explained to participants. All data was deidentified, and unique IDs were assigned to each participant; only the researcher or clinician at each site kept that information locked on their site).

All participants were compensated for their participation. They received US $100 for each of the 5 clinic visits (totaling US $500). At Site 1, participants received an additional US $100 for study completion. Site 2 decided not to make this final payment to stay within the US $500 IRS limits. As stated before, clinic visits with standard-of-care were provided at no cost, as well as the ability to monitor themselves with the home device. Given the amount of time involved in daily measurements, compensation for their time was recommended by the researchers.

### Procedure

**Figure 1.**
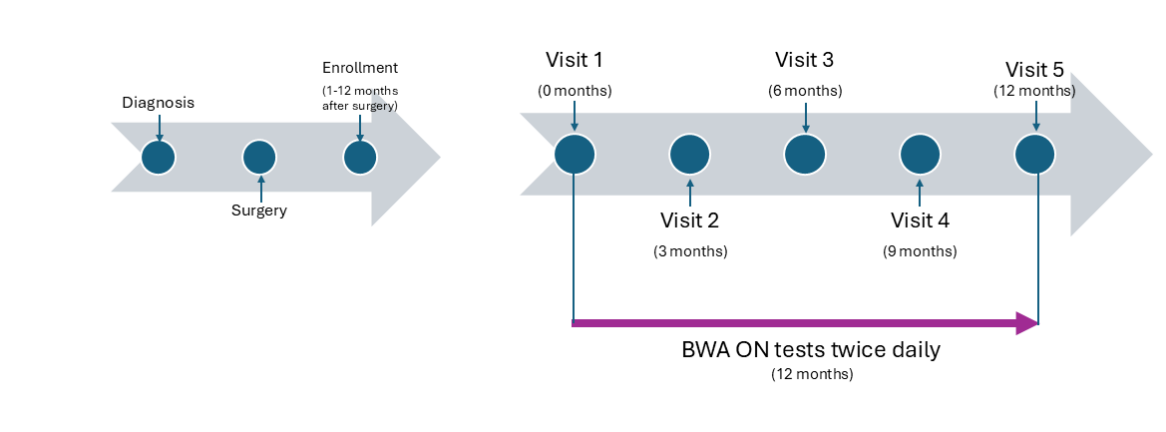
Timeline before study initiation and during the study.

A total of five clinic visits were scheduled at months 0 (the first visit upon enrollment), 3, 6, 9, and 12 months. The following measurements were recorded at each clinic visit: limb volume using tape measurement, BIA, using an InBody professional device (InBody 770; InBody Co. Ltd., Seoul, Korea, via InBody BWA) that has been used for research and clinic [12], range of motion, and quality of life assessments using DASH (The Disabilities of the Arm, Shoulder and Hand) and LLIS (Lymphedema Life Impact Scale), following the standard of care procedure. The clinician also examined the patient for any visible inflammation and asked if they were experiencing any discomfort. According to all the information, they determined the patient’s lymphedema stage from 0 to 2 and recommended follow-up treatment, if needed. Figure 1 shows a timeline of the study for reference, with the time before enrollment, starting from cancer diagnosis, surgery and enrollment and continuing with study visits and testing with the BIA home device specified.

To allow each participant to test themselves at home with a home-use BIA device, the BWA ON (InBody Co. Ltd., Seoul, Korea, via InBody BWA) was provided to them during their first clinic visit. This home BIA device uses 8-touch electrodes to apply small alternating electrical currents to the body at 3 different frequencies (5, 50, and 250 kHz). The BWA ON uses the same technology as professional InBody devices, validated against different gold standards. Devices were validated to DEXA (Dual-Energy X-ray Absorptiometry) by the Mayo Clinic, showing a concordance correlation coefficient (CCC) of 0.98 for fat-free mass (FFM) (5% limits of agreement: −3.5 to +5.2) and 0.97 for percent body fat (PBF) (5% limits of agreement: 6.0 to +4.2). The total body water (TBW) was validated in multiethnic populations against D2O (Deuterium Oxide Solution), showing that it produces valid estimates. Although some home BIA monitoring devices have been used previously [13], to the best of our knowledge, this is the first time that a home device has been available to patients for assessing segmental fluid levels.

The clinicians helped participants with setting up the device, assisted with the first measurement, and ensured the appropriate phone app (Apple or Google) was installed and working on the patient’s phone. The participant was registered by the clinician and data sharing with the clinic monitoring software was established.. Participants measured themselves daily: 30 minutes to 1 hour after waking and after 5 PM, before going to bed. Through the Apple or Google app, participants had access to their body composition (eg, weight, skeletal muscle, fat, and visceral fat level) and their extracellular-to-total body water (ECW/TBW) ratio levels. Clinicians were also able to monitor their progress remotely.

**Figure 2.**
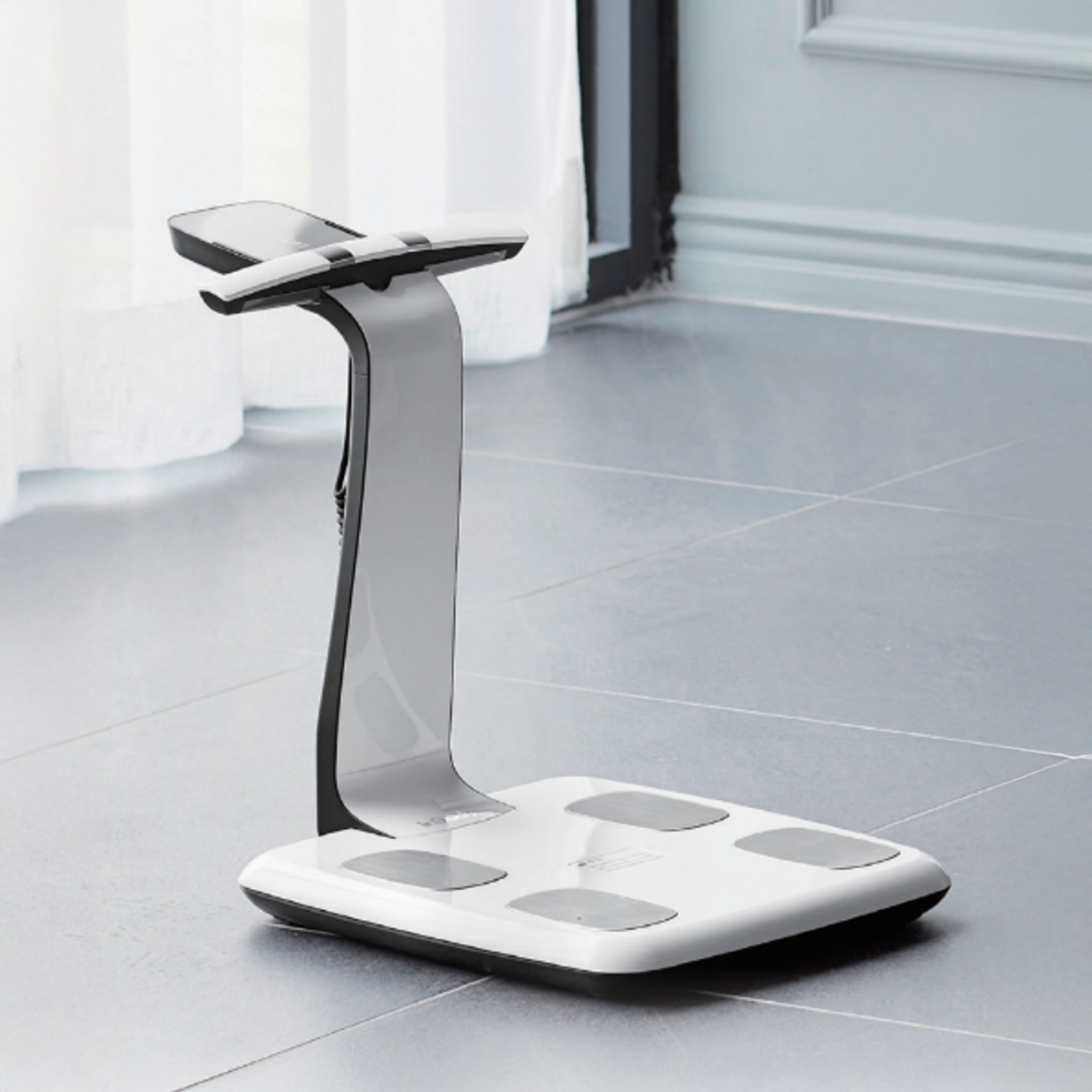
Bioelectrical body water analyzer for home use, BWA ON (InBody Co. Ltd., Seoul, Korea, via InBody BWA).

Figure 2 shows the home device used by participants at home. The black screen indicated the devices’ connection to the phone app via Bluetooth, and the patient could initiate the test. The phone app checked the electrode connections and started the test, showing its progress. Once the test was completed, patients were able to add details about exercise or any issues that may help explain detected changes in fluid levels or share if there was nothing new to report.

**Figure 3.**
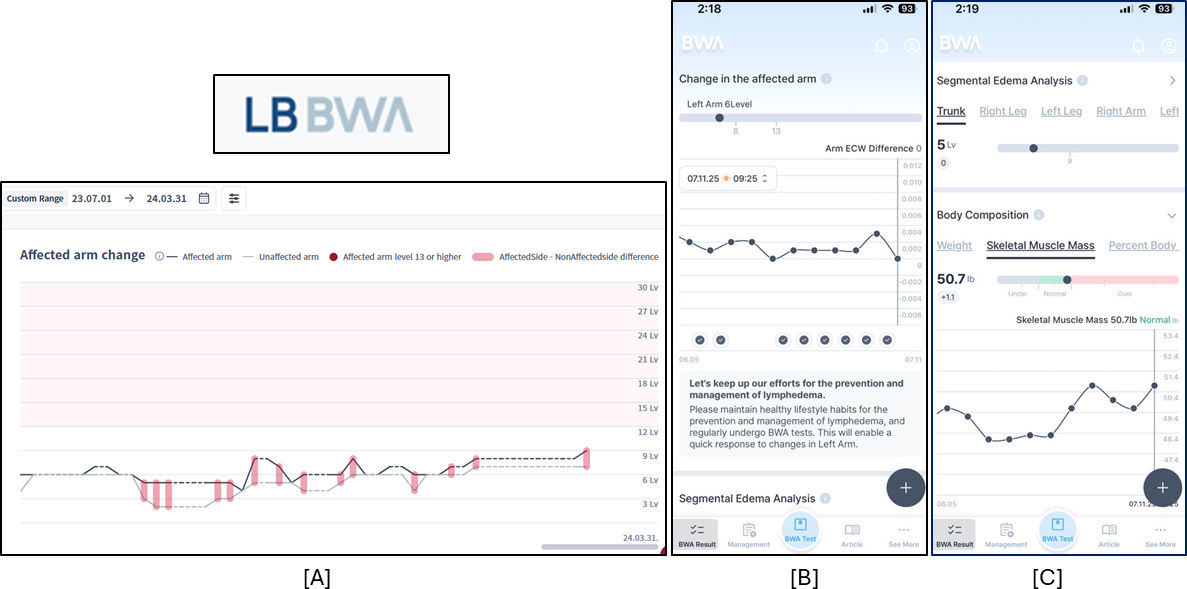
(A) Information displayed by the clinicians software and the patient’s Apple (or Google) phone app (B) ECW/TBW ratio difference between the two arms is shown in graph mode, (C) segmental ECW/TBW ratio is provided as level for limbs and trunk, and other body composition values (and history) are shown.

Figure 3 shows screenshots of the information available to participants in the Apple (or Google) app, as well as what the clinicians may see in the computer application to monitor their patients’ day-to-day fluid levels.

At the end of the study, participants were asked a few questions to assess the ease of use of the home device. They answered questions regarding daily use and the measurements provided.

## Results

Table 1 shows detailed demographic characteristics, surgery dates, and additional treatments required before or during the study, as these factors could affect water levels without lymphedema onset.

**Table 1.** Participants’ detailed demographics.

Case number	Age (yrs)	Dominant arm (A/L/R)^a^	Diagnosis date	Site	Surgery Date	Type	SLNB^b^ or ALND^c^		Radiation (Y/N)^d^ (B/D)^e^	Chemotherapy (Y/N)^d^ (B/D)^e^
100	66	A	01/24/23	L	01/30/23	Lumpectomy	SLNB		Y-B	N
101	66	R	02/23/23	L	03/21/23	Lumpectomy	SLNB		Y-D	N
102	64	R	02/07/23	L	03/30/23	Lumpectomy	SLNB		Y-D	N
105	52	R	02/20/23	L	03/20/23	Lumpectomy	SLNB		Y-D	N
110	54	R	02/14/23	L	03/10/23	Lumpectomy	SLNB		Y-D	N
112	54	R	04/21/23	L	06/14/23	Lumpectomy	SLNB		Y-D	Y-D
113	52	R	11/19/22	R	08/02/23	Lumpectomy	SLNB		Y-D	Y-B
200^f^	60	R	10/01/22	1. L 2. R	1. 03/10/23 2. 06/15/23	1. Lumpectomy 2. Mastectomy	1. ALND 2. n/a^g^		1. Y-D 2. n/a	1. Y-D 2. n/a

a (A/L/R): Ambidextrous/Left/Right.

b SLNB: Sentinel Lymph Node Biopsy.

c ALND: Axillary Lymph Node Dissection.

d (Y/N): Yes/No.

e (B/D): Before the study/During the study.

f 200 was the only participant to undergo prophylactic mastectomy.

g n/a: Not applicable.

Previous studies have shown that the prevalence of lymphedema diagnosis among patients undergoing breast cancer surgery is expected to be approximately 21.4% [14]. With eight cases presented in this initial report, only one case of lymphedema—or none—may have developed.

A nonpermanent change in water levels was detected in one participant undergoing chemotherapy treatment. Water levels returned to baseline once the treatment was completed, which coincides with previous reports of chemotherapy causing temporary swelling [15].

Fluid movement during the day could affect fluid distribution. This study evaluated the ECW/TBW ratio difference between the two arms (affected minus unaffected) in the morning and evening measurements and the interlimb (ILB) ECW/TBW ratio (affected arm/unaffected arm).

**Figure 4.**
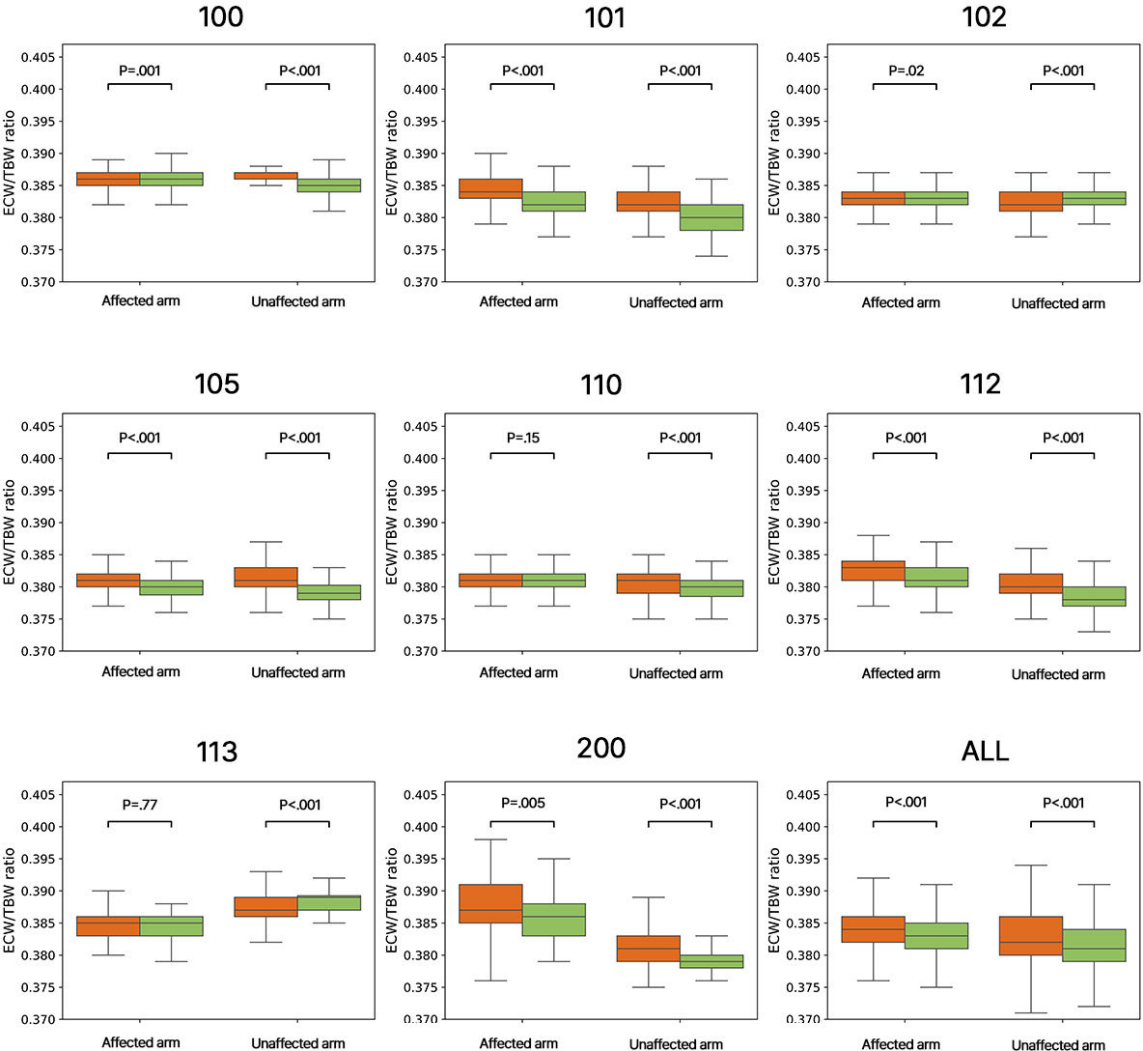
Morning, AM (red) and Evening, PM (green) ECW/TBW ratio measurements of the affected and unaffected arm. The bar plot shows the distribution of all values, and the *P* value of the comparison between the two measurements is shown above to determine if the difference between morning (AM) and evening (PM) measurements is significant. ECW/TBW: extracellular to total body water.

The daily ECW/TBW ratio measurements for the morning (AM) and evening (PM) are presented for the affected and unaffected arm in Figure 4. A Mann-Whitney U-test was performed on the data to determine if there was a significant difference between AM and PM measurements for each arm for each participant. AM and PM measurements were shown to be significantly different, except for 110’s and 113’s affected arms. A Rank-biserial correlation to determine effect size was used. Although values were small, most of them are positive, showing that AM values (red) of ECW/TBW ratio are higher than PM values (green), except for both arms for participant 102 and for 113’s unaffected arm, which showed that ECW/TBW ratio AM (red) values were lower than PM (green) values (case 100 and 102 Affected, have an opposite direction to what would be expected from the value shown in the table, due to rounding). This information is presented in Table 2, which also shows the number of tests performed by each participant in the AM and PM. Some participants are missing measurements in both the AM and PM, but in most cases, PM measurements were less frequent than AM measurements.

**Table 2.** ECW/TBW^a^ ratio values and number of measurements for each participant in the morning (AM) and evening (PM).

Case number and arm	ECW/TBW measurements (morning, AM), mean(SD)	Morning (AM) tests, n	ECW/TBW measurements (evening, PM), mean (SD)	Evening (PM) tests, n	*P* value Mann-Whitney *U*-test	Rank biserial correlation, *r*
100		341		314		
Affected	0.386 (0.0013)		0.386 (0.0016)		.001	0.132
Unaffected	0.387 (0.0012)		0.385 (0.0016)		<.001	0.507
101		356		317		
Affected	0.384 (0.0019)		0.382 (0.0023)		<.001	0.467
Unaffected	0.382 (0.0023)		0.380 (0.0026)		<.001	0.457
102		335		319		
Affected	0.383 (0.0020)		0.383 (0.0018)		.02	−0.093
Unaffected	0.382 (0.0021)		0.383 (0.0018)		<.001	−0.142
105		202		124		
Affected	0.381 (0.0046)		0.380 (0.0020)		<.001	0.344
Unaffected	0.381 (0.0049)		0.379 (0.0024)		<.001	0.432
110		313		191		
Affected	0.381 (0.0036)		0.381 (0.0020)		.15	0.064
Unaffected	0.380 (0.0030)		0.380 (0.0020)		<.001	0.222
112		365		350		
Affected	0.383 (0.0048)		0.381 (0.0028)		<.001	0.273
Unaffected	0.380 (0.0025)		0.378 (0.0054)		<.001	0.337
113		315		120		
Affected	0.385 (0.0024)		0.385 (0.0028)		.77	0.014
Unaffected	0.387 (0.0028)		0.389 (0.0024)		<.001	−0.229
200		200		83		
Affected	0.388 (0.0044)		0.386 (0.0039)		.005	0.166
Unaffected	0.381 (0.0028)		0.379 (0.0025)		<.001	0.227
All patients						
Affected	0.384 (0.0039)	2427	0.383 (0.0030)	1818	<.001	0.144

a ECW/TBW: extracellular to total body water.

All participants, except for 113, demonstrated at least slightly higher ECW/TBW ratios in the affected arm compared to the unaffected arm, both AM and PM. One potential difference between this participant and the other participants is that participant 113’s diagnosis and surgery occurred in the dominant arm. Lymphedema literature has found different cutoffs for dominant and nondominant arms’ volume, and BIA impedance ratio [6], so this could help explain this result. Participant 200 shows the largest interarm difference.

**Figure 5.**
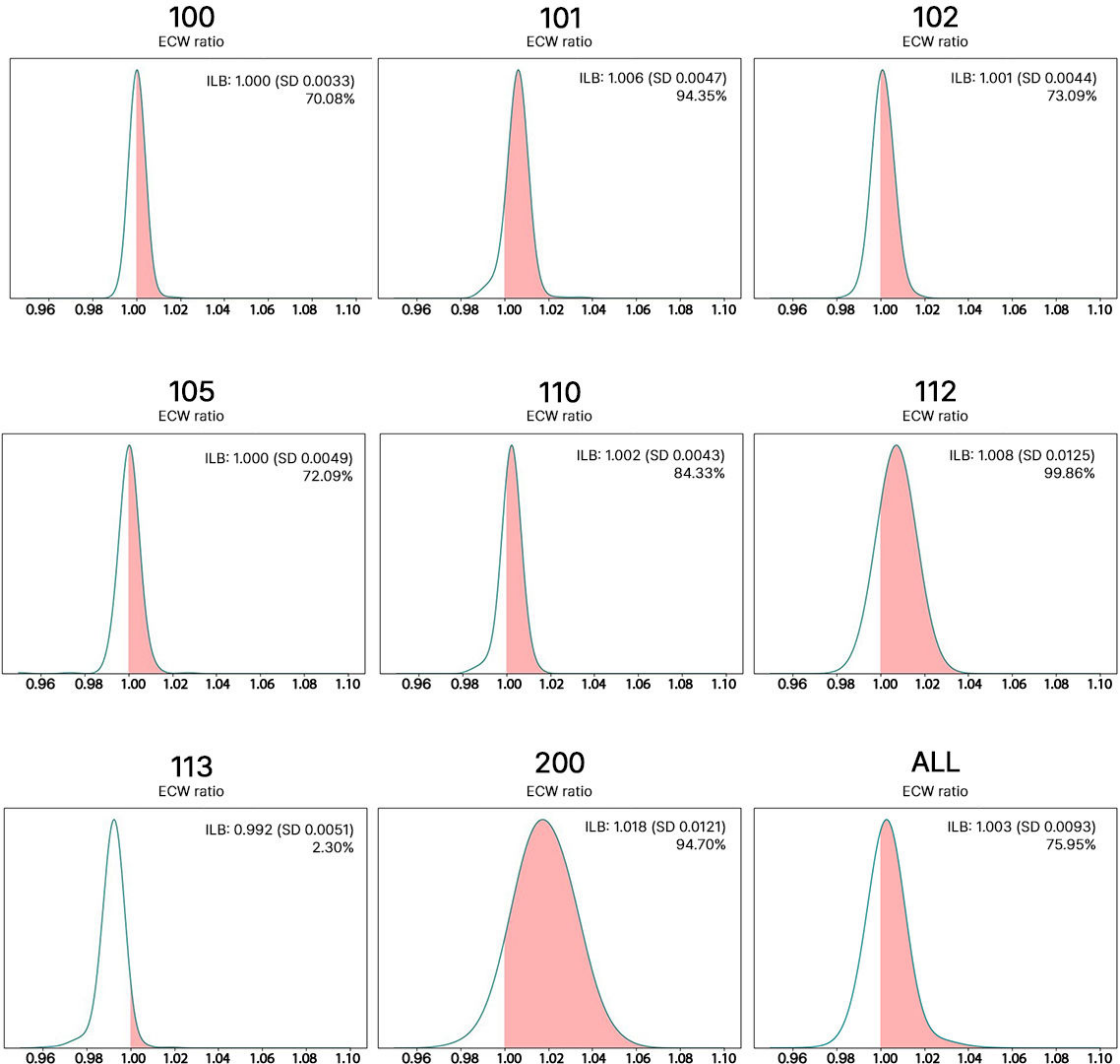
Distribution of the interlimb ratio (ILB) of the ECW/TBW ratio of the affected and unaffected limb larger than ‘1’ (orange). The average value and standard deviation of the interlimb ratio are shown along with the percentage of times the ratio is larger than 1 (below). ECW: extracellular water; TBW: total body water; ILB:interlimb; ECW ratio: ECW/TBW ratio

As seen in Figure 5, all participants have an interlimb (ILB) value ([ECW/TBW_affected arm_]/[ECW/TBW_unaffected arm_]) larger than ‘1’ at some point, meaning that the ECW/TBW ratio in the affected arm is larger than the ratio in the unaffected arm. Ideally, the arms are balanced, and the value is ‘1’. Others have shown that a ratio close to 1 is related to lymphedema onset [16-18]. Considering all participants, occurrences of ILB larger than ‘1’ took place in 75.95%. Participants 101, 110, 112, and 200 show a higher percentage (>80%), with 112 being the highest. Treatments during the study (Table 1) could affect fluid levels, so this average may not confirm lymphedema but may help determine if those changes are permanent or not. Occurrences of values larger than 1 are below average for 100, 102, and 113. Further analysis is needed to determine if they underwent less radiation/chemotherapy treatment.

Although not significantly different, all participants showed a decrease in weight and ECW/TBW ratio in both arms by the end of the study.

At the end of the study, 7/8 participants agreed that the device was easy to use daily, and the measurements were easy to understand. A few participants had some issues that were solved either by replacing the device or by troubleshooting, but they all agreed that they felt there was a benefit to having the home device to use.

## Discussion

### Principal Findings

Significant differences between AM and PM fluid levels measurements were found, which indicates the need to establish a proper protocol to follow up patients, either in the morning or afternoon to reduce their burden. More data would be needed to determine if the ECW/TBW ratio difference, as well as the ratio between the affected and unaffected arms, is also affected by the time of day. This would help confirm if measurements need always to be conducted in AM and PM, or if only one measurement, one time during the day, may suffice when monitoring bilateral differences (unilateral lymphedema risk). In this study, we only considered unilateral lymphedema risk when a risk of bilateral lymphedema exists, tests may also have to be conducted at the same time of the day, always, as there would not be a contralateral reference limb (unaffected) to compare to, but a follow-up study would be needed to determine this.

Although one participant (113) whose affected arm was the dominant arm showed a different behavior compared to those whose affected arm was the nondominant arm, additional data will be needed to confirm that the BWA ON data is affected the same way as has been shown by previous literature [12], with lymphedema cutoff values being different.

Cases where treatment, such as radiotherapy or chemotherapy, resulted in significant changes in ECW/TBW or substantial differences between the two arms were reported. Possible BCRL was reported using the home BIA device for participant 200, as the ECW/TBW ratio between the affected and unaffected arms reached cutoff values (around 1.03, with small differences in each paper, which also depends on the population being used in the study - different stages) defined by previous literature [16,17,19].

### Comparison to Previous Work

This study aimed to verify whether it is feasible to use a home BIA device that monitors fluid levels (BWA ON) with patients at risk of developing breast cancer-related lymphedema. Traditionally, patients visit the hospital every 3 to 6 months for lymphedema assessment. However, daily home monitoring has not been available, and patients have not been able to be tested both in the AM and PM to determine if there may be significant differences in measurements, which may affect lymphedema assessment. Home-based BIA monitoring is meaningful as it could quickly alert patients and clinicians to fluid changes.

### Strengths and Limitations

This is the first study in which patients could objectively monitor fluid levels at home and relate those measurements to their subjective symptoms of discomfort or tightness. This allowed them to reach out to the therapist and discuss any changes they may have seen, being more proactive with their care. Additionally, participants expressed that they felt they benefited from using the device.

This study proved that it is feasible for patients to use the home device while allowing clinicians to monitor them without having to visit the clinic.

The first limitation of this study was the small number of participants, which did not allow for further analysis, such as comparing participants who developed lymphedema to those who did not, as only one participant appeared to have developed the disease. This is a case report to determine the feasibility of following participants at home, so no additional participants were included.

Second, the morning and evening home measurements provided an additional burden to participants, besides their necessary treatment, which caused at least one participant to withdraw from the study earlier. AM and PM measurements were necessary to determine possible differences between times of day. In practice, the goal is to reduce patient burden by encouraging testing themselves once at the same time of day.

### Future Work

Increasing the number of participants monitoring themselves daily and with the professional BIA device in the clinic would help determine the correlation with other traditional methods (eg, tape measurements, range of motion, therapists’ assessments) of determining lymphedema to aid therapists in making treatment decisions.

Currently, lymphedema assessment considers different measurements, including BIA in many cases. Staging lymphedema is an even more complex process that also depends on the therapist’s concept and experience. In the future, a large study that considers staging may be helpful to improve patient outcomes. It may also help guide patients better when using a home monitoring device, to empower them more to take care of their health.

### Conclusion

Lymphedema is a well-known disease that is difficult to improve after irreversible symptoms affect quality of life. The ECW/TBW ratio of the upper extremities, measured through BIA, could be an indicator of the development and severity of BCRL [18]. Preliminary data suggest that daily at-home BIA monitoring is feasible, which in the future may aid early detection and prevention by empowering patients as well as clinicians.

## Abbreviations

BCRL: breast cancer related lymphedema
BIA: bioelectrical impedance analysis
ECW: extracellular water
TBW: total body water
ECW/TBW: extracellular to total body water
ECW ratio: ECW/TBW ratio
ILB: interlimb
